# Viral Etiology of Common Cold in Children, Finland

**DOI:** 10.3201/eid1502.081468

**Published:** 2009-02

**Authors:** Aino Ruohola, Matti Waris, Tobias Allander, Thedi Ziegler, Terho Heikkinen, Olli Ruuskanen

**Affiliations:** Turku University Hospital, Turku, Finland (A. Ruohola, T. Heikkinen, O. Ruuskanen); University of Turku, Turku (M. Waris); Karolinska University Hospital, Stockholm, Sweden (T. Allander); National Public Health Institute, Helsinki, Finland (T. Ziegler)

**Keywords:** Common cold, etiology, rhinovirus, enterovirus, adenovirus, human bocavirus, human metapneumovirus, coronavirus, influenza virus, children, Finland, letter

**To the Editor**: The common cold is regarded as a viral disease. In the first years of the 21st century, several new respiratory viruses have been identified, such as human metapneumovirus (hMPV), coronaviruses NL63 and HKU1, and human bocavirus (HBoV). Many studies have addressed the role of these viruses in hospital settings, but few studies have been conducted among outpatients. We examined the etiology of the common cold in young children who were newly symptomatic but had no need of hospital care. We hypothesized that the etiology could be detected in all cases by using modern diagnostics that test for 16 viruses in outpatients.

Between February 1996 and April 1998, we collected nasopharyngeal aspirate samples in an outpatient setting from 194 Finnish children having newly onset (<48 h) symptoms of common cold but no acute otitis media (AOM) or other symptoms demanding antimicrobial therapy (*1*). The mean age of the study population was 2.1 years (range 0.7–3.9 years), and 81% attended day care. The parents of all participants gave written informed consent, and the study protocol was approved by the Ethics Committee of Turku University Hospital in Turku, Finland.

The nasopharyngeal aspirate samples were processed freshly for antigen detection (respiratory syncytial virus [RSV]; parainfluenza viruses 1, 2, and 3; influenza A and B viruses; and adenovirus) by time-resolved fluoroimmunoassay (*2*). Stored samples were subjected to nucleic acid testing (NAT) for picornaviruses; RSV; coronaviruses 229E, OC43, NL63, and HKU1; influenza C virus; HBoV; hMPV; and adenovirus. Recently, these samples were reanalyzed for rhinovirus and enterovirus using real-time PCR for the amplification step (*1*,*3*–*6*).

At least 1 respiratory virus was detected in 179 (92%) of 194 children. Rhinovirus was the most common respiratory virus, found in 138 (71%) children (Table). Other viruses were found in varying proportions: HBoV was present in 27 (14%) children; adenovirus was found in 23 (12%) (3 were positive by antigen detection, and 23 by NAT); enterovirus was present in 20 (10%); coronaviruses were found in 11 (6%) (NL63:7; HKU1:2; 229E/OC43:2); influenza viruses were present in 11 (6%) (A:4; B:1; C:6); RSV was shown in 8 (4%) (all were positive by antigen detection and NAT); parainfluenza viruses were present in 7 (4%) (1:1; 3:6); and hMPV was found in 3 (2%). The Table shows the concomitant occurrence of all viruses. Among children with a positive viral finding, 46 (26%) had 2 viruses, and 10 (6%) had 3 or 4 viruses concomitantly. The viruses occurring most frequently with other viruses were adenovirus (100%), HBoV (81%), and enterovirus (75%).

**Table:** Positive viral findings in 194 children with newly onset uncomplicated common cold, Finland, 1996–1998

Virus	No. (%) positive*
Rhinovirus	91 (47)
Rhinovirus and human bocavirus	13 (7)
Rhinovirus and adenovirus	11 (6)
Rhinovirus and enterovirus	6 (3)
Human bocavirus	5 (3)
Enterovirus	5 (3)
Respiratory syncytial virus	5 (3)
Influenza C virus	4 (2)
Parainfluenza virus 3	4 (2)
Rhinovirus, adenovirus, and enterovirus	3 (2)
Coronavirus NL63	2 (1)
Human metapneumovirus	2 (1)
Coronavirus 229E or OC43	2 (1)
Rhinovirus and parainfluenza virus 3	2 (1)
Rhinovirus and influenza A virus	2 (1)
Human bocavirus and enterovirus	2 (1)
Adenovirus and enterovirus	2 (1)
Rhinovirus, adenovirus, and coronavirus NL63	2 (1)
Rhinovirus, human bocavirus, adenovirus, and enterovirus	2 (1)
Influenza A virus	1 (1)
Influenza B virus	1 (1)
Coronavirus HKU1	1 (1)
Rhinovirus and respiratory syncytial virus	1 (1)
Rhinovirus and coronavirus NL63	1 (1)
Rhinovirus and parainfluenza virus 1	1 (1)
Human bocavirus and respiratory syncytial virus	1 (1)
Human bocavirus and coronavirus NL63	1 (1)
Human bocavirus and influenza C virus	1 (1)
Adenovirus and respiratory syncytial virus	1 (1)
Coronavirus NL63 and influenza A virus	1 (1)
Rhinovirus, human bocavirus, and influenza C virus	1 (1)
Rhinovirus, adenovirus, and human metapneumovirus	1 (1)
Rhinovirus, human bocavirus, adenovirus, and coronavirus HKU1	1 (1)
Total positive	179 (92)
Total negative	15 (8)
Total children sampled	194 (100)

*Percentages rounded to nearest whole number.

Although our diagnostic panel was incomplete, lacking parechoviruses and parainfluenza type 4 virus, we detected >1 respiratory viruses in 92% of the children who had a common cold. As expected, rhinovirus was the leading cause of the common cold in these children. The role of picornaviruses was also emphasized by the abundance of enteroviruses. Enterovirus has gained attention mainly in severe infections, e.g., meningoencephalitis, and is rarely included in diagnostics for respiratory infections. However, PCR has shown that enterovirus commonly causes upper and lower respiratory infections that may be complicated by AOM or expiratory wheezing (*4*,*7*). Thus, enterovirus should be included in the diagnostic panels of respiratory infections. HBoV was the second most prevalent virus in our study population. Since its discovery in 2005, HBoV positivity has been reported in 3%–19% of different study populations (*8*). Its pathogenic role has been questioned because most HBoV cases are co-infections with other viruses (*8*), and 81% of those testing positive for HBoV in our study had co-infections. However, adenovirus and enterovirus reached similar co-infection frequencies, likely because of prolonged postinfection viral shedding of these agents. HBoV–specific immunoglobulin (Ig) M and IgG antibody responses were recently reported in children with wheezing, suggesting that HBoV induces a systemic infection and is probably a true causative agent of lower respiratory tract disease (*9*). Our study indicates that HBoV may also be a common cause of common cold in young children. However, we found hMPV, coronaviruses NL63 and HKU1, and influenza C virus in 1%–4% of the children, suggesting that these viruses play a minor role in childhood common cold. Our study may underestimate the role of RSV and hMPV because we excluded children with AOM, which is frequently related to these viruses.

Multiple viral findings were common in our study, and 3 children had 4 viruses concomitantly, a logical finding because young children are constantly exposed to respiratory viruses, especially if they attend day care. A recent follow-up study showed that almost all viral findings were related to symptoms, thus supporting the argument that most, if not all, viruses are causative agents (*10*).

A possible causative agent of the common cold can be found in nearly all children who have a cold, and rhinovirus is the leading causative agent. In our study, HBoV was also found frequently, but the recently discovered viruses hMPV and coronaviruses NL63 and HKU1 played a minor role in the common cold of young children.
